# Reddish Colour in Cooked Ham Is Developed by a Mixture of Protoporphyrins Including Zn-Protoporphyrin and Protoporphyrin IX

**DOI:** 10.3390/foods11244055

**Published:** 2022-12-15

**Authors:** Claudia Giménez-Campillo, Juan de Dios Hernández, Isidro Guillén, Natalia Campillo, Natalia Arroyo-Manzanares, Carlos de Torre-Minguela, Pilar Viñas

**Affiliations:** 1 Department of Analytical Chemistry, Faculty of Chemistry, Regional Campus of International Excellence “Campus Mare Nostrum”, University of Murcia, 30100 Murcia, Spain; 2 Department of Research and Development, PROSUR S.A.U., Av. Francisco Salzillo, P/27-2, San Ginés, 30169 Murcia, Spain

**Keywords:** natural pigments, protoporphyrins, Zn-protoporphyrin IX, cooked ham colour

## Abstract

The nitrosyl–heme complex is considered the pigment responsible for the development of reddish colour in cooked hams. However, the same reddish colour was observed in a nitrite-free product elaborated with polyphenols, suggesting the presence of other red pigments that can contribute to generate this colour. In this study, the protoporphyrins composition of the pigment solution obtained from nitrite and nitrite-free cooked hams was analysed using 80% (*v/v*) acetone/water solution for extraction. Chromatographic analysis using a combination of diode array and fluorescence detectors revealed the presence of protoporphyrin IX and Zn-protoporphyrin IX in this solution, and these protoporphyrins were subsequently identified with complete certainty by mass spectrometry. These results show how the colour of cooked hams can be developed by a mixture of different protoporphyrins and also demonstrate the absence of selectivity of acetone/water extraction for measuring the content of nitrosyl–heme in cooked hams.

## 1. Introduction

The colour of cooked hams is a critical parameter in establishing their quality and is a key factor that consumers use to make their selection. In this sense, nitrites have been used for a long time in the elaboration of cooked hams as an ingredient in brine, which allows the meat product to acquire a pink–red colour together with specific flavour and antibacterial protection [1]. However, the classification of nitrites and nitrates as compounds with probably carcinogenic activity in humans by the International Agency for Research on Cancer (IARC) has led to a reduction or even substitution of their use in the production of processed meat [2]. As an alternative to nitrites, the addition of polyphenols as ingredients in the brine allows the development of red colour in cooked hams, and it has been proposed as a substitute of nitrites in their production. The evaluation by CIE L*a*b* technique showed that the colour obtained in a product made with nitrite is similar to that developed in a product made with polyphenols (NATPRE T-10 HT S) [3], raising the question of whether nitrosyl–heme is the only red pigment responsible for the colour of cooked ham.

Protoporphyrins are red porphyrin-derived pigments that have four modified pyrrole units and two propionic acid groups. Protoporphyrin IX (PPIX) contains four methyl groups, two vinyl groups, and two propionic acid groups. This compound is found in nature in the form of complexes, with the two inner hydrogen atoms replaced by a metal cation. When complexed with the iron(II) cation, the molecule is named heme (FePPIX), being a prosthetic group in some proteins such as haemoglobin and myoglobin. PPIX can also react with other metal ions, such as zinc(II), to give zinc protoporphyrin IX (ZnPPIX) or with iron salts to give the complex FeCl(PPIX) known as hemin. 

In the meat industry, the spectrophotometric method developed by Hornsey is well accepted to determine the concentration of nitrosyl–heme complex as the main porphyrin pigment responsible for the red colour observed in cooked ham treated with nitrites [4]. This method is based on an extraction considered selective of this heme-derived compound with acetone/water solvent. Remarkably, in this study, we obtained a similar spectral pattern among the red pigments obtained from hams made with nitrites or with polyphenols in the absence of nitrites, which suggests a lack of selectivity in the pigment extraction procedure. In this regard, it should be pointed that dry-cured hams develop red colour in absence of nitrites or other additives, and ZnPPIX was identified as the main donor of red colour in these meat products, as is the case of Iberian and Parma hams [5,6]. In addition, ZnPPIX can be extracted from Parma ham using a 75% (*v/v*) acetone/water solution and shows a strong fluorescence [5,7]. 

The aim of this study was to further analyse the pigments extracted from cooked hams made with or without nitrites using the same procedure described by Hornsey. The analysis was carried out using an analytical platform based on high-performance liquid chromatography (HPLC) coupled to diode array detection (DAD), fluorescence detection (FLD), and quadrupole time-of-flight mass spectrometry (QTOF-MS). Herein, we report the presence of three protoporphyrins (FePPIX, ZnPPIX and PPIX) in the pigment mixture obtained from these cooked hams, independently of the content of heme groups present in the muscle tissue used in their elaboration. Therefore, these results reject the idea of a selective extraction of nitrosyl–heme complex using an acetone/water solution and suggest a role of other protoporphyrins, including ZnPPIX, in the development of colour in cooked hams.

## 2. Materials and Methods

### 2.1. Reagents and Standards

Individual protoporphyrin standards (FeCl(PPIX), ZnPPIX and PPIX) were obtained from Sigma Aldrich (St. Louis, MO, USA), with purities between 90 and 97%. The standard of FePPIX (94.65% purity) was obtained from LGC standards (Middlesex, UK). Each week, standards were prepared at 1000 μg mL^−1^ in N,N-dimethylformamide (DMF) which was provided by J.T. Baker (Gliwice, Poland). In addition, dilutions with DMF were prepared each day to work with due to the instability of the compounds. The dilutions were kept in the refrigerator at 4 °C and in the dark because they are photosensitive compounds. 

Organic solvents such as methanol, acetonitrile and acetone were supplied by Chem-Lab (Zedelgem, Belgium). Sodium chloride, sodium nitrate, sodium ascorbate and ammonium acetate were supplied by Sigma Aldrich and Fisher Chemical (Madrid, Spain). Polyphenol-rich extract was obtained from PROSUR S.A.U. (Murcia, Spain), commercialized as NATPRE T-10 HT S (20% of total polyphenols).

### 2.2. Cooked Hams and Sample Preparation

Four types of cooked hams were elaborated for this study. Two of them were made with two different batches of pig shoulder meat (High Heme group) and two others were made with two different batches of pig hind leg meat (Low Heme group). Each of the types of ham was prepared independently. In all cases, the meat was ground (CATO, Girona, Spain) to a size of 12 mm and was mixed with its corresponding brine ingredients in a vacuum mixer (CATO, Girona, Spain) mixed for 2 h under vacuum conditions. The resulting meat–brine mixture was stuffed in sausage casing and cooked until reaching 68 °C core temperature (maximum oven temperature was 73–75 °C). 

Five cooked hams were elaborated with a brine containing 1.8 g of NaCl, 0.012 g of NaNO_2_ and 0.03 g of sodium ascorbate per 100 g of pig shoulder meat to obtain hams with 120 mg kg^−1^ of NaNO_2_ (High Heme (nitrite)) from the same lot of ground meat. On the other hand, a cooked ham was made with the same proportions of brine ingredients but with pig hind leg meat (Low Heme (nitrite)). In addition, five nitrite-free cooked hams were made with a brine containing 1.4 g of NaCl and 1 g of polyphenol rich extract (NATPRE T-10 HT S) per 100 g of pig shoulder meat (High Heme (NATPRE)) from the same lot of ground meat. A control cooked ham was elaborated in the same conditions as before but without NaNO_2_ or polyphenol-rich extract and with pig hind leg meat (Low Heme (0 ppm nitrite)).

Five g of minced ham sample was homogenized with a solution elaborated with 20 mL of acetone and 1.5 mL of water, at 5000–7000 rpm with a polytron homogenizer (IKA, t25 digital Ultraturrax, Staufen, Germany) for 2 min. After leaving 10 min at 4 °C, the mixture was centrifuged (FC5706, OHAUS, Germany) at 1620× *g* for 5 min at room temperature protected from day light. The supernatant containing the pigments extracted was filtered with 1 mL of needle-free Nipro Syringes and nylon filters (25 mm, 0.45 µm) (Agilent Technologies) and protected from day light. The absorbance of each supernatant was scanned in a UV spectrophotometer (MultiGO, Thermo Fischer (Waltham, MA, USA)) ranging from 300 to 700 nm with 1 nm of spectral bandwidth.

### 2.3. HPLC-DAD-FLD

An Agilent 1100 HPLC apparatus (Agilent, Waldbronn, Germany) equipped with a quaternary pump (G1311A), a degasser (G1379A) and coupled to dual detection by diode array (G4212B) and fluorescence (G1321A) detectors was used for the analysis of samples. A volume of 20 µL of each sample was injected using an automatic injector (G1329A) at a flow rate of 1 mL min^−1^. An ACE Excel 3 C18 column (150 × 4.6 mm, 3 µm, Symta (Madrid, Spain)) was used for red pigments separation with a mobile phase composed of 0.01 M ammonium acetate (solvent A) and methanol (solvent B) applying a gradient elution. The gradient elution was 75% B 0 min, 75% B 2.5 min, 100% B 2.8 min, 100% B 8 min, 75% B 8.4 min and 75% B 10 min. The wavelengths selected in the diode array detector (DAD) were 400 nm and 540 nm; meanwhile, the wavelengths selected in the fluorescence detector (FLD) were 410 nm (excitation) and 590 nm (emission).

### 2.4. UHPLC-QTOF-MS

An Agilent 1290 Infinity II Series HPLC system (Agilent Technologies, Santa Clara, CA, USA) was used to analyse the samples. An analytical ZORBAX RRHD Eclipse Plus C18 column (100 × 2.1 mm, 1.8 μm, Agilent Technologies (Diegem, Belgium)) with an in-line filter of 0.3 µm (Agilent Technologies) was used for pigments separation, which was kept at 25 °C. The injection volume for all the samples was 20 µL, and the same gradient elution conditions that were previously used in Section 2.3 were applied, with a flow rate of 0.4 mL min^−1^. The detector was an Agilent 6550 Q-TOF mass spectrometer (Agilent Technologies), with an Agilent Jet Stream Dual electrospray source (AJS-Dual ESI) working in positive ionization mode and with the following operating parameters: capillary voltage, 4000 V; nebulizer gas pressure, 30 psi; drying gas flow, 16 L min^−1^; drying gas temperature, 130 °C; fragmentor voltage, 350 V; nozzle voltage, 500 V and 1 RF Vpp octapole, 750 V. The mass range used to record the exact mass spectra was 50–1100 *m/z*. 

From the total ion chromatograms (TICs), the exact mass measurements of each peak were obtained using an automated calibration to correct the masses and, in the 50–1100 *m/z* range, MS/MS data were obtained by performing 3 fragmentation experiments at 0, 10 and 40 V collision energy. The MassHunter Qualitative Analysis Navigator Agilent Technologies software, version B.08.00, and MS-DIAL software, version 4.80 [8], was used to analyse the data obtained. 

The extracted ion chromatogram (EIC) of the protonated molecule of each analyte was used with the exception of FeCl (PPIX), which was in the non-protonated form. From the molecular formula of the compounds, the exact theoretical masses were obtained from the ChemSpider website and the molecular mass calculator tools of MassHunter software. The exact mass spectra of the analytes were obtained by subtracting the EIC background. Each analyte was confirmed and quantified using its exact mass. The peak areas of the EICs were used to quantify the analytes. Table 1 shows the UHPLC-QTOF-MS settings to monitoring ions of the target analytes as well as their retention times, the precursor ions (*m/z*) and their corresponding instrumental error in ppm, which was calculated as the difference between the experimental and theoretical *m/z* values divided by the theoretical *m/z* value and multiplied by 10^6^.

### 2.5. Statistical Analysis

A Mann–Whitney U test was applied for two-group comparisons. Data processing, statistical analysis and graphics were performed using Prism software (Graph-Pad Software, Inc., San Diego, CA, USA). The *p* value is indicated as *p* < 0.05; *p* > 0.05 not significant (ns).

## 3. Results and Discussion

**Figure 1:**
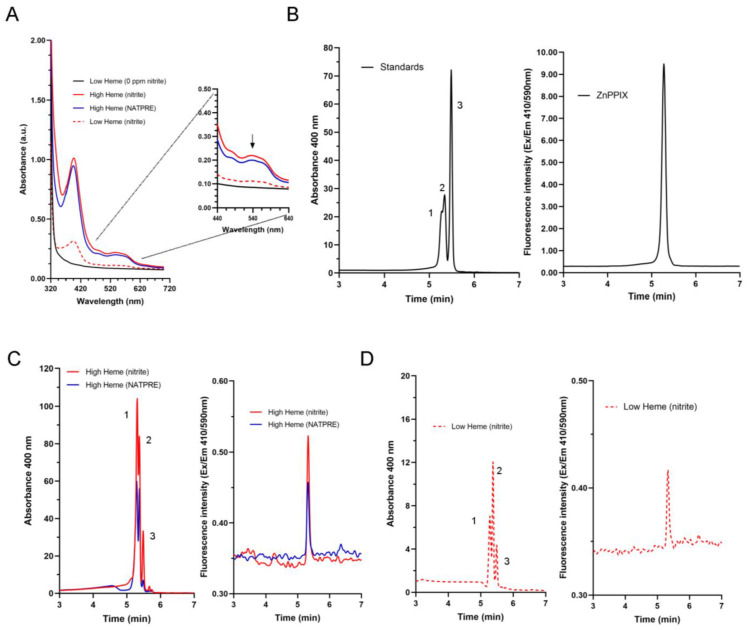
Analysis of pigments extracted from different cooked hams with 80% (*v/v*) acetone/water solution by HPLC-DAD-FLD. (**A**) Visible absorption spectra of pigments extracted from the studied cooked hams. Maximum absorbance at 540 nm is indicated with an arrow. (**B**) HPLC elution profile of a standard mixture of protoporphyrins (1 µg mL^−1^): 1, FePPIX; 2, ZnPPIX and 3, PPIX. (**C**) HPLC elution profile of pigments extracted from cooked hams made with high heme content muscles. (**D**) HPLC elution profile of pigments extracted from cooked hams made with low heme content muscles.

The absorption spectra obtained from the solution of red pigments extracted from cooked hams with an acetone/water solution, either made with nitrites or with a nitrite-free alternative (NATPRE T-10 HT S), show identical profiles with two peaks of maximum absorbance at 400 nm (Soret band) and 540 nm (Figure 1A). These cooked hams were made from pig shoulder, which is a tissue that contains a high concentration of heme group [9]. These results suggest a similar extraction of the protoporphyrin pigments involved in the colour of cooked ham in both cases, despite the fact that one type of ham was made without nitrite. In addition, we show the absorption profile of the red pigments extracted from cooked hams elaborated with pig hind leg, a tissue with low content of heme group, indicating that a lower heme content in cooked hams elaborated with nitrites only reduces the absorbance values but does not modify the absorbance profile of the pigments involved in meat colour. In contrast, a cooked ham elaborated without nitrites (0 ppm) does not show absorbance at these wavelength ranges nor does it have the desired reddish colour (Figure 1A).

Protoporphyrin standards were separated and identified by HPLC using a combination of DAD and FLD. The chromatographic conditions were optimized using different mobile phases based on mixtures of methanol or acetonitrile with water or acetic acid/ammonium acetate buffers. Best results were obtained by using 0.01 M ammonium acetate as solvent A and MeOH as solvent B. Then, it was tested to work with different gradients or using isocratic conditions, obtaining better results with the following gradient: 75% B 0 min, 75% B 2.5 min, 100% B 2.8 min, 100% B 8 min, 75% B 8.4 min and 75% B 10 min. All protoporphyrin standards were detected at 400 nm, while ZnPPIX was also identified as a single peak by FLD operated at 410 nm and 590 nm as excitation and emission wavelengths, respectively (Figure 1B). 

The chromatographic analysis of the pigment solutions obtained from the cooked hams studied revealed the presence of all these protoporphyrins regardless of heme group content. The profiles obtained using the DAD detector at a wavelength of 400 nm showed several peaks appearing at the same retention times obtained for FeCl(PPIX), ZnPPIX and PPIX (Figure 1C,D). Moreover, ZnPPIX was also identified as a single peak by FLD at the same retention time as the standard (Figure 1C,D). These data demonstrate a lack of specificity of the extraction method based on acetone/water developed to measure the nitrosyl–heme complex [4], and therefore, we cannot establish that the nitrosyl–heme complex is the only porphyrin pigment responsible for the red colour observed in cooked ham. Moreover, our results indicate that the spectrophotometric method commonly used in the meat industry is not suitable for evaluating the presence of nitrosyl–heme in cooked hams, requiring the application of more specific methods such as the measurement by mass spectrometry of the nitrous oxide generated from nitric oxide released from nitrosyl–heme in acidic conditions [3,10].

**Figure 2:**
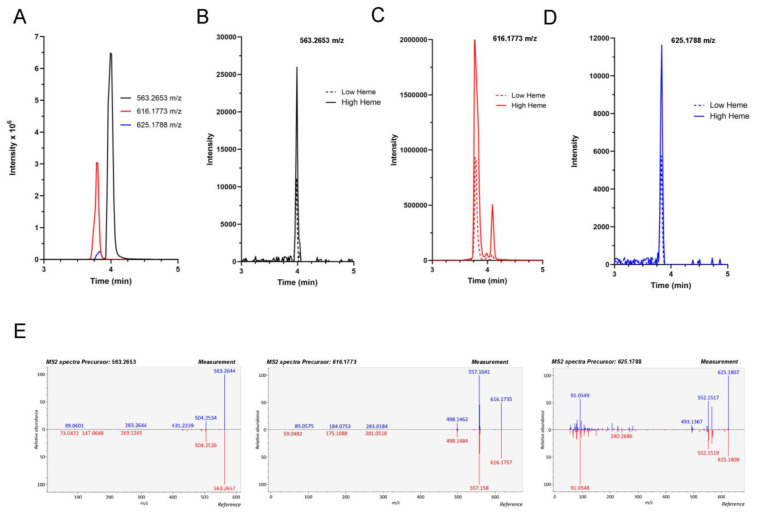
Analysis of pigments extracted from different cooked hams with 80% (*v/v*) acetone/water solution by UHPLC-QTOF-MS. (**A**) Extracted ion chromatograms obtained for a standard mixture of protoporphyrins (100 ng mL^−1^). (**B**) Extracted ion chromatogram of 563.2653 *m/z*; (**C**) 616.1773 *m/z* and (**D**) 625.1788 *m/z* obtained from pigments extracted from cooked hams made from high heme muscle or low heme muscle. (**E**) Deconvoluted MS/MS spectrum and spectra matching results of PPIX (left), FePPIX (centre) and ZnPPIX (right) yielding dot-product scores of 0.984, 0.954 and 0.960, respectively.

In an attempt to achieve the better identification of the protoporphyrins extracted from the cooked ham samples, the pigment solutions were analysed by UHPLC-QTOF-MS in positive mode to achieve unequivocal identification of the compounds. Previously, we determined the precursor ion of each protoporphyrin (563.2653 *m/z* PPIX, 616.1773 *m/z* FePPIX and 625.1788 *m/z* ZnPPIX) analysing a standard mixture of protoporphyrins in the same chromatographic conditions (Figure 2A). The extracted ion chromatograms (EIC) obtained from the pigment samples allow us to conclude the three protoporphyrins studied are present in them (PPIX (Figure 2B), FePPIX (Figure 2C) and ZnPPIX (Figure 2D)). The presence of all of these protoporphyrins in the analysed samples regardless of the heme content of the studied cooked ham is demonstrated. Finally, an identification with complete certainty for all protoporphyrins studied in the samples was obtained by comparing the exact masses, the HRMS/MS fragmentation spectra and retention times of chromatographic standards (Figure 2E).

The validation of HPLC-DAD and HPLC-FLD methodologies was carried out by obtaining the calibration curves, linearity ranges, limits of detection (LODs) and limits of quantification (LOQs), and precision for the protoporphyrins (Table 2). The selectivity of the FLD detector was higher for ZnPPIX, since FePPIX is not detected by fluorescence and the peak corresponding to ZnPPIX can be integrated more accurately. For this reason, we applied HPLC-FLD methodology for the quantification of ZnPPIX, although this protoporphyrin showed similar sensitivity with both detectors. The rest of the protoporphyrins were quantified using HPLC-DAD, obtaining adequate RSD values in all cases.

Finally, the amount of the different protoporphyrins identified in samples of cooked ham elaborated with nitrites or with a nitrite-free alternative (NATPRE T-10 HT S) was analysed (Table 3). The results pointed to no significant differences in the concentration of FePPIX and ZnPPIX in the cooked hams studied (Mann–Whitney U test *p* > 0.05); however, we observed a significant decrease in the concentration of PPIX in the cooked hams elaborated with a nitrite-free alternative (NATPRE T-10 HT S) (*p* = 0.012).

Zn-PPIX was detected in all the analysed samples, even in samples with NATPRE T-10 HT S (Zn-free). Zn-PPIX is a compound found naturally in the cells involved in the synthesis of Fe-PPIX as a final step of their biosynthetic pathway in the mitochondria. After complete Fe-PPIX synthesis, PPIX could be chelated with zinc by mitochondrial enzyme ferrochelatase reducing the excess of PPIX [11,12]. 

## 4. Conclusions

The chromatographic analysis developed in this study has revealed the presence of other protoporphyrins such as PPIX and ZnPPIX in the acetone/water extracts used to evaluate the red pigments involved in the colour development of cooked hams, suggesting that these protoporphyrins also contribute to the reddish colour observed. In addition, our results demonstrate that the traditional spectrophotometric method used to measure nitrosyl–heme in cooked hams is not suitable due to the lack of specificity of acetone extraction.

## Figures and Tables

**Table 1:** Analytical parameters for HPLC-QTOF-MS.

Protoporphyrin	RT, min	Formula	*m/z* Theoretical	*m/z* Experimental	Error (ppm)
PPIX	3.90	C_34_H_32_N_4_O_4_Na_2_	563.2653	563.2650	0.53
FePPIX/FeCl(PPIX)	3.69	C_34_H_32_FeN_4_O_4_/C_34_H_32_ClFeN_4_O_4_	616.1773	616.1765	1.30
ZnPPIX	3.76	C_34_H_32_ZnN_4_O_4_	625.1788	625.1769	3.04

**Table 2:** Validation parameters for protoporphyrins calibration using HPLC-DAD-FLD.

Protoporphyrin	Linear Range ng g^−1^	Linearity R^2^	LOD ^1^ ng g^−1^	LOQ ^2^ ng g^−1^	RSD ^3^ %
HPLC-DAD					
PPIX	30–35,000	0.998	17.0	26.3	9.2
FeCl(PPIX)	60–35,000	0.997	18.1	59.8	10.4
ZnPPIX	75–35,000	0.992	22.6	74.7	9.7
HPLC-FLD					
ZnPPIX	90–35,000	0.994	26.3	86.9	8.6

^1^ LOD: Limit of detection. ^2^ LOQ: Limit of quantification. ^3^ RSD: Relative standard deviation (*n* = 10).

**Table 3:** Concentration of protoporphyrins in individual cooked hams made with nitrites or with a nitrite-free alternative (NATPRE T-10 HT S).

Sample Number	FePPIX (DAD) ng g^−1^ ham	ZnPPIX (FLD) ng g^−1^ ham	PPIX (DAD) ng g^−1^ ham
High Heme (nitrite) 1	29,162	148	2105
High Heme (nitrite) 2	19,629	171	5909
High Heme (nitrite) 3	10,678	157	3070
High Heme (nitrite) 4	10,086	169	4123
High Heme (nitrite) 5	31,931	119	1503
High Heme (NATPRE) 1	22,671	124	992
High Heme (NATPRE) 2	24,547	117	643
High Heme (NATPRE) 3	22,600	113	392
High Heme (NATPRE) 4	25,052	122	147
High Heme (NATPRE) 5	8333	159	1205

## Data Availability

Data is contained within the article.
